# GP registrar well-being: a cross-sectional survey

**DOI:** 10.1186/1447-056X-9-2

**Published:** 2010-02-09

**Authors:** Peter Schattner, Dennis Mazalin, Ciaran Pier, Jo Wainer, Mee Yoke Ling

**Affiliations:** 1 Department of General Practice, Faculty of Medicine, Nursing and Health Sciences, Monash University, Building 1, 270 Ferntree Gully Rd, Notting Hill Vic 3168, Australia; 2 School of Psychology, Deakin University, 221 Burwood Highway, Burwood Vic 3125, Australia; 3 Monash Institute of Health Services Research, School of Public Health and Preventive Medicine, Faculty of Medicine, Nursing and Health Sciences, Locked Bag 29, Clayton 3168, Australia

## Abstract

**Objectives:**

To investigate the major stressors affecting GP registrars, how those at risk can be best identified and the most useful methods of managing or reducing their stress.

**Design, setting and participants:**

Cross-sectional postal questionnaire of all GP registrars in one large regional training provider's catchment area.

**Main outcome measures:**

The Depression, Anxiety and Stress Scale (DASS), a specifically developed Registrar Stressor Scale consisting of five subscales of potential stressors, plus closed questions on how to identify and manage stress in GP registrars.

**Results:**

Survey response rate of 51% (102/199). Rural difficulties followed by achieving a work/life balance were the principal stressors. Ten percent of registrars were mildly or moderately depressed or anxious (DASS) and 7% mild to moderately anxious (DASS). Registrars preferred informal means of identifying those under stress (a buddy system and talks with their supervisors); similarly, they preferred to manage stress by discussions with family and friends, debriefing with peers and colleagues, or undertaking sport and leisure activities.

**Conclusions:**

This study supports research which confirms that poor psychological well-being is an important issue for a significant minority of GP trainees. Regional training providers should ensure that they facilitate formal and informal strategies to identify those at risk and assist them to cope with their stress.

## Introduction

A significant number of Australian general practitioners (GPs) experience high levels of stress and have poor psychological health, and this is likely to be due to a combination of individual characteristics and environmental factors such as frequent exposure to work-related stressors [1-3]. In recent times there have been changes to the Australian medical workforce and GP training, with a decline in the number of registrars and an apparent gradual attitudinal shift away from the rigid medical 'martyrdom' of previous generations toward better 'work/life balance'[4,5]. These changes may make stress as important an issue as it is among GP registrars' more senior colleagues. Stress might be related to specific training issues as well as simply working in general practice.

This questionnaire survey aimed to identify causes and potential responses to stress within one major regional training provider (RTP). The principal areas of research interest were: 1) What are the major stressors that affect GP registrars in relation to their work and training? 2) What are the most effective ways of identifying which GP registrars are most at risk of stress? 3) What are the most useful methods of managing and reducing GP registrar stress?

## Methods

### GP registrar questionnaire development

The questionnaire was developed following a literature review and interviews with 6 registrars. It was piloted with two recent medical graduates to establish face and content validity. These graduates indicated that the questionnaire could be completed within fifteen minutes. The questionnaire comprised four sections: demographics, the Depression, Anxiety Stress Scale (DASS), a Registrar Stressor Scale (RSS) and questions on identifying and managing stress.

The RSS was developed by the research team and covered five subscales representing the main stressors which had been identified through the literature review and the interviews. These were: rural difficulties (e.g. finding accommodation, separation from family), isolation/low social support (e.g. not having time for developing new social networks), work conditions (e.g. excessive workload, difficulties in making complaints about training), insufficient teaching/vocational support, and work/life balance. The RSS items had four response choices using a Likert scale. Registrars were also asked to separately indicate one or more items in rank order to identify the factors contributing the most to registrar stress, the strategies they normally use to cope with stress and what they thought were the most effective ways of identifying registrars who experience significant stress. They were also asked about options for identifying and managing stress.

The final component of the questionnaire was the 21-item version of the *Depression Anxiety Stress Scale (DASS) *[6]. The DASS consists of three subscales designed to assess depression, anxiety, and stress

### Procedure

The survey was conducted within a large metropolitan RTP and sent out to registrars in all levels of the program (excluding the six who participated in the interviews). Registrars were in their hospital, basic, subsequent and advanced terms of training, including metropolitan and rural rotations.

The RTP mailed the questionnaires, explanatory information and reply-paid envelopes to 199 registrars. The envelopes, which were addressed to the research team, were coded so that non-responders could be followed up, with the RTP sending a reminder email to non-responders after two weeks.

### Statistical analysis

The results of the survey are presented with descriptive statistics and with differences between sub-groups of respondents tested by analysis of variance (ANOVA). An hierarchical regression analysis was conducted to examine the relative strength of particular subscales of the RSS in predicting stress, as measured by the total score on the DASS. SPSS version 17 was used for data analysis.

### Ethics Clearance

The Standing Committee on Ethics in Research involving Humans (SCERH) at Monash University granted ethics approval for the study.

## Results

### Response rate and demographics

A total of 102 GP registrars (females = 67, males = 35) completed the questionnaire, yielding a response rate of 102/199 or 51 percent. The mean age of the sample was 33.10 (SD = 6.36), with a range of 25 to 51 years.

The GP registrars were in the following stages of their training: subsequent term (42.2%); advanced term (32.4%); basic term (10.8%); hospital term (9.8%); and rural term (2.0%). On average, the GP registrars had spent 1.93 years (SD = 1.11) in the training program.

Participants reported the following living arrangements: with a partner/spouse (66.7%); with a friend(s) (10.8%); living alone (10.8%); and living at home with a parent(s) (6.9%). Thirty-nine percent of registrars report having a child in their care (49% have one child, 44% have two children, and 7% have three or more children). Seventeen percent were international medical graduates (IMGs), and 60% had completed or were currently completing the rural term.

Twenty-eight percent of registrars reported a history of psychiatric/emotional problems (depression = 65%, stress = 12%, emotional problems = 12%, anxiety = 8%, and psychosis = 3%). Ten registrars reported having a secondary mental health problem, that is, in addition to their primary one. Secondary problems included anxiety (8), depression (1) and psychosis (1). Seventy-nine percent of GP registrars stated that they had their own GP.

### Causes of stress

**Figure 1:**
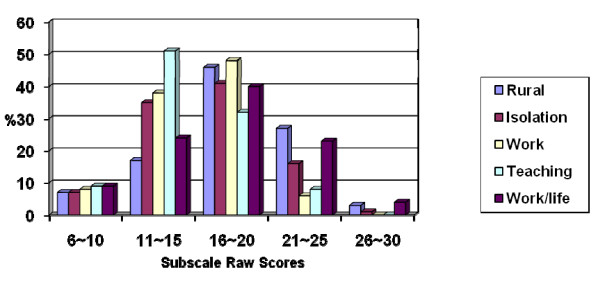
**Percentages of raw scores in categories on the RSS subscales**.

Each subscale on the RSS raw score ranges up to a maximum of 30. The higher the mean score, the greater the impact level. These RSS raw scores are presented in Figure 1 in five evenly distributed categories. Although for most subscales the greatest number of participants rated the stressors in the 16 to 20 raw score category, about a quarter (27% and 23% respectively) of the registrars rated the rural difficulties and work/life balance stressors in the higher 21 to 25 raw score category. These two issues were seen to be the main stressors faced by registrars (Table 1).

**Table 1:** Levels and causes of stress: means (and standard deviations) for the DASS and the RSS subscales

Subscales (DASS and RSS)	Mean (SD)
Depression (DASS)	6.33 (7.00)

Anxiety (DASS)	3.40 (4.31)

Stress (DASS)	10.80 (7.64)

Total (DASS)	20.43 (15.73)

Rural difficulties (RSS)	18.30 (4.41)

Isolation/low social support (RSS)	16.20 (4.30)

Work conditions (RSS)	15.60 (3.14)

Insufficient teaching/social support (RSS)	15.00 (3.82)

Work/life balance (RSS)	17.10 (4.88)

### Anxiety, depression and stress based on the DASS

Lovibond and Lovibond have created cut-off scores for the subscales of the DASS, allowing the scores to be rated for severity across five categories: normal, mild, moderate, severe, and extremely severe [6]. For each of the three subscales (i.e. depression, anxiety and stress), the majority of GP registrar scores were in the normal category. Approximately 10% of registrars reported mild or levels of depression and stress, another 10% were moderate, and 7% reported mild or moderate levels of anxiety respectively. Very few of the GP registrars were in the 'severe' and 'extremely severe' categories on any subscale (Table 2).

**Table 2:** Percentage of GP registrars in each DASS category

*Subscale*	*Normal*	*Mild*	*Moderate*	*Severe*	Extremely Severe
Total Sample (*N *= 102)					

Depression	73.50 (0-9)	9.80 (10-13)	10.80 (14-20)	4.00 (21-27)	2.00 (28+)

Anxiety	83.30 (0-7)	6.90 (8-9)	6.90 (10-14)	2.00 (15-19)	1.00 (20+)

Stress	73.50 (0-14)	9.80 (15-18)	10.80 (19-25)	5.00 (26-33)	1.00 (34+)

*Note*. Figures in parentheses denote the Lovibond and Lovibond (1995) DASS severity rating cut-offs for each category.

### Group Differences across the RSS and the DASS

Female GP registrars reported the following subscales of the RSS to be significantly greater stressors than for male GP registrars: Work conditions (females: mean = 16.20, SD = 2.75; males mean = 14.50, SD = 3.55; F(1, 100) = 7.10, p = 0.009, partial eta squared = 0.07); Work/life balance (females: mean = 18.16, SD = 4.54; males mean = 15.00, SD = 4.90; F(1, 100) = 10.57, p = 0.002, partial eta squared = 0.10); and Rural difficulties (females: mean = 19.12, SD = 4.28; males mean = 16.42, SD = 4.21; t(58) = -2.29, p < .05). There were no significant differences on the RSS between IMGs and non-IMGs, or between registrars who had completed the rural term and those who had not. There were no significant group differences (males/females, IMGs/non-IMGs, and completed rural term/not completed rural term) on the DASS scales.

### Specific factors that contribute most to registrar stress

The Registrar Questionnaire asked registrars to indicate which individual factors in their training and work contributed most to their stress, and to rank order the factors they had chosen, from the most stressful to the least stressful (Table 3). Of the fifteen factors presented, the three that were most often ranked as number one were: managing tasks in limited time frames (24%); difficult patients (19%), and difficulties associated with the rural term (15%). The order of results from columns one and two are similar, i.e. the stressors faced by the greatest proportion of respondents were also those most commonly ranked as being the most stressful.

**Table 3:** Factors that have contributed most to stress

Percentages nominated for stress factors	1	2
Difficult patients	75	19

Managing tasks in limited time frames	67	24
GP fellowship exams	52	10

Starting as a basic registrar	47	5

Difficulties associated with the rural term	43	15

Transition from hospital to independent GP work	39	6

Negotiating work or training conditions	35	3

Session times when you are the only doctor working in the practice	32	1

Isolation from others	32	2

Financial restraints	31	3

Long working hours	30	3

Different computer programs when beginning at a new practice	28	1

Inadequate training support structures	20	1

Inadequate support from the practice	12	1

Bullying from other staff members	10	2

*Note*. 1 = the percentage of registrars endorsing this stress factor; 2 = the percentage of registrars overall who ranked the stress factor as the number one stress factor.

### Methods of identifying GP registrar stress

Table 4 shows that the preferred methods of identifying registrar stress were: a buddy system (26%); interviews with your GP supervisor (21%), and informal means (e.g., staff noticing changes in demeanour) (13%).

**Table 4:** Most effective methods of identifying registrar stress

Percentages nominated for each method	1	2
Interviews with your GP supervisor	58	21

A buddy system	57	26
Seeing your own GP	53	9

Informal means (eg., staff noticing changes in demeanour)	46	13

Interviews with a VMA representative (eg., psychologist)	35	10

Stress screening on the annual GP registrar satisfaction survey	34	7

Psychological paper and pencil test	26	6

Chat room forum facilitating discussion about registrar stress	23	3

*Note*. 1 = the percentage of registrars endorsing this method; 2 = the percentage of registrars overall who ranked the method as the number one method.

### Predicting GP registrar stress

A hierarchical regression analysis was conducted to examine the relative strength of subscales of the Registrar Stressor Scale in predicting stress, as measured by the total score on the DASS. The Insufficient teaching/vocational support subscale, and Age, were entered on the first step and Work conditions and Work/life balance subscales were entered on the second to ascertain whether they were able to predict a significant amount of variance on the DASS scores. The results are presented in Table 5. Insufficient teaching/vocational support was shown to be a significant predictor at step one but Age was not shown to be significant. The step one model explained 12 percent of the variance. Following step 2, Work conditions (beta = .29) and Work/life balance (beta = .26) were both found to be significant predictors of stress, with the former the stronger one. The ANOVA table indicates that the model, which explained 27 per cent of the variance, was significant [F(4, 97) = 8.90, p < .001]. This result suggests that negative work conditions and difficulties with balancing work and life commitments are greater predictors of stress for GP registrars than the teaching program, vocational support or age.

**Table 5:** Summary of hierarchical regression analysis for variables predicting stress

	Beta Weights
**Step1**	

Insufficient teaching	.35*

Age	-.05

**Step 2**	

Insufficient teaching	.06

Age	-.06

Work conditions	.29***

Work/life balance	.26***

*Note*. Insufficient teaching = Insufficient teaching/vocational support subscale; beta weights = standardized beta coefficients; R Squared = .12 for Step 1; R Squared Change = .15 for Step 2 (*p *< .001). *p < .001. **p < .01. ***p < .05.

### Assisting registrars to manage or reduce their stress

Seventy five per cent of the GP registrars reported that the RTP should be actively involved in identifying which registrars are stressed. The three strategies that were most often ranked as the number one most effective means of managing stress by registrars include: debriefing with peers/colleagues (34%); education sessions on identifying/coping with stress (18%); and regular organised sport or fitness activity (9%). Debriefing with peers/colleagues was reported as an effective strategy significantly more often by females than males *χ*^2 ^(1) = 6.30, *p *< .05, and was reported significantly more often by local graduates compared to IMG's *χ*^2 ^(1) = 19.48, *p *< .001. No other group differences were found (Table 6).

**Table 6:** The most effective strategies for managing GP registrar stress

Coping Strategies	1	2
Debrief with peers/colleagues	78	34

Seeing a clinician	55	7
Regular organised leisure activities	53	5

Education sessions on identifying/coping with stress	50	18

Regular organised sport or fitness activity	49	9

Relaxation training (eg., meditation)	40	4

Facilitate greater support from family or peers	43	8

Time management course	42	2

A targeted approach, addressing the specific problems	39	8

A buddy system focussing on immediate strategies	38	6

Online or face-to-face support groups	31	4

Easy to use self-help book with handy tips	25	1

*Note*. 1 = the percentage of registrars endorsing this strategy; 2 = the percentage of registrars overall that ranked the strategy as the most effective.

About half the registrars in this study had contemplated leaving medicine as a result of stress, and more than a third reported that occupational stress had made them want to leave their current workplace. Approximately one quarter are thinking about leaving the training program or general practice.

## Discussion

### The extent of psychological disturbance among GP registrars

This survey showed that for the majority of GP registrars, depression, stress, and anxiety levels were in the normal range. However, 11% experienced moderate levels of depression and stress and 6% experienced severe depression and stress, which is of concern and should warrant action. Moderate levels of depression as indicated by the DASS screening tool are often significant enough to warrant a clinical diagnosis [6]. By comparison, a major survey of mental health among the Australian population has found that the prevalence of all affective disorders among younger people (aged 18 - 35) is between 6 and 7% [7].

The findings of the study suggest that occupational stress had a significant impact, with 50% of current GP registrars having thought, at least at some point in time, about leaving the medical profession due to stress related reasons. Rural term difficulties and problems with achieving a work/life balance were the general stressors with the greatest impact, with females significantly more affected than males. A study by Larkins et al also found both of these stressors to be of significant concern for Australian GP registrars, and others have also highlighted the many problems in the rural term which can lead to increased stress levels [8-12].

Significantly more females than males in the sample reported rural term difficulties, consistent with previous research into the negative effects of the rural placement on female GP registrars [13]. Importantly, previous research has indicated that improving the psychological well-being of rural GPs increases the likelihood of retaining them in rural practice [14].

Poor work/life balance has also been found to be an important stressor in studies of GPs, UK GP registrars, Australian GP registrars and Australian medical students [15-18]. Although some research indicates that work/life balance issues are equally disruptive for both female and male medical graduates, this study found that balancing these commitments was more of an issue for female GP registrars than males [19,20]. This is consistent with other research which has emphasised the 'balancing-act' between medicine and the additional work that women do in managing family commitments [11,21].

Investigation of the individual stress factors for GP registrars revealed that managing tasks in limited time frames and difficult patients were the highest and second highest ranking individual stress factors. Difficulties with such work conditions have reportedly contributed to a drop in GP registrar numbers in the UK and should therefore be carefully addressed by Australian training programs [22-24].

In addition, the findings from the work conditions subscale of the RSS revealed that this stressor was significantly greater among females than males. This highlights the suggestions by researchers such as McDonald et al on the need for mentorship, supervision, and training to be gender-specific because of the underlying masculine character of medical culture [12]. The third highest ranking individual stress factor was difficulties associated with the rural term, which reinforces its significant impact.

This study has examined the relationship between training and psychological distress, but has not explored the extent to which uniquely individual and pre-existing personality factors contribute to coping difficulties. Caution must be applied in assuming cause-and-effect relationships in individual cases, although the overall results for this group of registrars remains of some concern. Further, although the survey asked specifically about training issues, much of the stress is likely to be bound up with the work of being a GP rather than a registrar; it is not really possible to separate these two states. Although any training program will inevitably have some degree of stress, this survey suggests levels that are higher than many would accept as reasonable.

### Identifying which GP registrars are most at risk of stress

Recent research has shown that as psychological problems increase among Australian GPs, their help-seeking decreases [25]. This suggests that identifying GP registrar stress may be difficult. The GP registrars' reported preference for informal assessment of their stress suggests a lack of confidence in more formal systems such as consultation with a GP, or psychological screening.

The identification of GP registrar stress may also be aided by the better understanding of which situations or factors are predictive of registrar stress. Difficult work conditions and problems with balancing work/life commitments were shown to be greater predictors of stress for GP registrars than the teaching program/vocational support or the age of registrars. This is in line with the Larkins studies which found that workload, the negotiation of work terms and conditions, and feeling powerless to influence change at work were particularly associated with registrar stress [26,27].

### Assisting GP registrars to manage and reduce their stress

The stress reduction strategies most frequently used by the respondents include talking to family or friends, debriefing with peers or colleagues, and sport or exercise. These strategies involve dialogue with other people and appear to be problem-focused coping strategies. These are typically more adaptive in the long-term than emotion-focused strategies such as avoidance or distraction [27]. This study could not ascertain whether these registrars deliberately under-reported less acceptable strategies such as alcohol or substance use. The fourth most reported strategy was just putting up and getting on with it, which would suggest some level of emotion-focused coping.

The majority of the participants reported that the RTP should be involved in helping them manage their stress levels. The most effective stress reduction strategies were identified as debriefing with peers/colleagues, education sessions on identifying/coping with stress, and regular organised sport or fitness activity. Again, the first two methods are problem-focused and adaptive in nature [27]. The strategies address stress reduction from a combination of approaches: involving colleagues, learning more about stress and how to approach it, and the physical and social benefits associated with organised sport or fitness. This finding provides clear guidelines for RTPs to address stress among their trainees.

### Limitations of the Study

The study had a 51% response rate which raises the question of response bias. Unfortunately, surveys among doctors tend not to have high response rates; this study is therefore not exceptional. Although the attitudes of non-responders cannot be known, it is reassuring that participants did arise from a reasonable cross-section of registrars according to gender and year of training. A second limitation is that the study relied on self-report by registrars who originate from one RTP, albeit a large one. Nevertheless, this survey is consistent with other research which indicates that improving GP registrar well-being remains an important issue for the future of general practice [28].

## Conclusion

Mental health difficulties are common in this group, with almost 30 percent of the respondents having reported a history of psychiatric or emotional problems. Ten out of the 28 reported a secondary mental health problem in addition to their primary one. Half the registrars in this study had contemplated leaving medicine as a result of stress, and more than a third reported that occupational stress had made them want to leave their current workplace, with approximately one quarter thinking about leaving the training program or general practice. Appendix 1 lists several recommendations which arise logically from the findings of this study which RTPs may wish to consider in order to improve the psychological health of their trainees.

## Appendix 1: Key recommendations to regional training programs

### List of recommendations arising from this study to regional training programs

1. GP registrar well-being remains an important and relevant issue for RTPs

2. The rural rotation continues to be problematic and further investigation on how this term can be made more satisfying should be undertaken.

3. RTPs should pay further attention to improvements in working conditions, including providing more flexibility in rosters and in time off.

4. Registrars need further education and support in learning how to manage tasks in a limited time, how to deal with difficult patients and how to cope with rural terms.

5. The use of a buddy system should be encouraged.

6. Registrars should be encouraged to talk to family and friends about their difficulties, and to debrief with colleagues rather than 'bottle things up'.

7. RTPs should facilitate regular discussions between registrars and their GP supervisors about the registrar's welfare and educate supervisors about the typical stressors encountered and how they can help registrars cope with these.

8. RTPs should inform practices, perhaps largely but not exclusively through the supervisors, about the need to be sensitive to the emotional health of registrars.

9. Where possible, RTPs should encourage registrars to engage in extra-curricular activities such as sport or other relaxing pastimes.

10. RTPs should include educational sessions in the training program curriculum on how to cope better with stress.

## Competing interests

The Victorian Metropolitan Alliance (a regional training program) funded the study which was conducted at Monash University (department of general practice). The RTP sent out the survey questionnaires but these were collected by the university department. The RTP did not have any role in designing the research methods or the questionnaire, or in data analysis and interpretation. There were no other competing interests.

## Authors' contributions

PS was the chief investigator and wrote the final version of the article; DM was the research officer, did most of the data analysis and wrote the first draft of the article; all authors were equally involved in the design of the study and in reviewing drafts of the paper. All authors read and approved the final manuscript.
